# Cross-chest liposuction plus gland resection through single minimal incision for management of grade I/II gynecomastia

**DOI:** 10.1186/s12893-025-02980-z

**Published:** 2025-07-03

**Authors:** Saber M. Abdelmaksoud, Ahmed Omran, Khaled Maher Muhammad Elagamy, Mahmoud Abdelhamid Elhendawy, Barakat Abdelreheem Mahmoud

**Affiliations:** 1 Plastic and Reconstructive Surgery Department, Port-Said University, Port-Said, Egypt; 2 Plastic and Reconstructive Surgery Department, Al-Azhar University, Damietta, Egypt; 3 General Surgery Department, Delta University for Science and Technology, Damietta, Egypt; 4 Plastic and Reconstructive Surgery Department, Al-Azhar University, Assiut, Egypt; 5 Plastic and Reconstructive Surgery Department, Helwan University, Cairo, Egypt

**Keywords:** Gynecomastia, Liposuction, Minimally invasive surgery

## Abstract

**Background and aim:**

In modern gynecomastia surgery, minimally invasive access and fast recovery have been advocated as essential management concepts. The present study aimed to describe the surgical details and clinical outcome of a minimally invasive approach combining cross-chest liposuction and minimal incision gland resection for management of grade I/II gynecomastia.

**Methods:**

The present retrospective study included 30 patients with grade I/II gynecomastia. Surgery was conducted under general anesthesia with the patient placed in the supine position and arms abducted 90^○^ at the chest. The whole surgical procedure was achieved through a minimal 1-cm stab incision made at the inferolateral areolar border. The incision was used for injection of tumescent solution, liposuction and gland excision. The operative outcome was assessed by both patients and independent surgeons. Evaluated aesthetic aspects included breast symmetry, nipple and areola shape, projection of the nipple-areolar complex (NAC), contour regularity, and overall appearance.

**Results:**

Postoperative complications included hematoma (3.3%) and hypopigmented scar (6.7%). Patient-reported scores for breast symmetry, nipple and areola shape, projection of the nipple-areolar complex, contour regularity and overall appearance were 4.2 ± 0.4, 4.3 ± 0.5, 4.3 ± 0.4, 4.1 ± 0.3 and 4.4 ± 0.5 respectively while surgeon-reported scores for breast symmetry, nipple and areola shape, projection of the NAC, contour regularity and overall appearance were 4.5 ± 0.5, 4.4 ± 0.5, 4.4 ± 0.5, 4.2 ± 0.4 and 4.1 ± 0.3 respectively.

**Conclusions:**

The combination of cross-chest liposuction and gland excision through a single minimal incision at the inferolateral areolar border provides good and satisfactory aesthetic outcomes for patients with grade I/II gynecomastia.

## Introduction

Gynecomastia is defined as persistent benign mammary gland enlargement in men which may be unilateral or bilateral [1]. The condition is quietly common with estimated prevalence of 32.3% according to one study [2]. The etiology can be primary due to hormonal imbalance without definitive underlying cause or secondary to medications, systemic diseases or malignancy [3]. There are four grades of gynecomastia depending on size of glandular hypertrophy and overlying skin ptosis [4]. Diagnosis is usually clinical but radiological confirmation using mammography, ultrasonography or magnetic resonance imaging may be required [5].

Gynecomastia is generally regarded as a benign condition with spontaneous regression expected in most patients. In some situations, pharmacological treatment may be tried. However, gynecomastia lasting more than 1 year tend to have fibrosis which makes medical treatment difficult and surgical management is the treatment of choice [6].

Surgical options vary according to grade of gynecomastia. Skin-sparing mastectomy with or without liposuction is the most frequently used approach followed by mastectomy with skin reduction [7]. The combination of surgical excision and aspiration techniques is suggested to reduce the rate of complications as compared to surgical excision alone [8].

Aesthetically, appropriate management of gynecomastia should provide almost complete glandular excision, proper position and shape of the nipple-areola complex and masculine reflection of the overlying skin [9]. In modern gynecomastia surgery, minimally invasive access, scar minimizing techniques [10], and fast recovery have been advocated as essential management concepts [11]. Outpatient surgery under local anesthesia was also suggested [12]. Minimal incision designs for glandular tissue removal have consistently improved patients’ satisfaction and quality of life [13]. However, many of these designs entailed use of more than one incision or required one incision with suboptimal aesthetic outcome. Few studies tried to improve the clinical outcome of gynecomastia surgery through combining the advantages of minimally invasive surgical access and enhanced liposuction techniques.

The present study aimed to describe the surgical details and clinical outcome of a minimally invasive approach combining cross-chest liposuction and minimal incision gland resection for management of grade I/II gynecomastia.

## Patients and methods

### Design, setting and ethics

The present retrospective study was conducted at Helwan and Al-Azhar University Hospitals. The study protocol was approved by the ethical committee of Al-Azhar Faculty of Medicine (Approval No.: DFM-IRB00012367-24–05–016) and all patients provided informed consent to use their anonymous clinical data for research purposes in accordance with the Helsinki Declaration on clinical research involving human subjects.

### Selection of patients

Thirty patients were included in the present study. They had grade I/II gynecomastia according to Simon grading [14]. Patients were excluded if their gynecomastia was related to associated liver disease or endocrine disorders or if they were previously submitted to breast surgery.

### Preoperative preparation

All patients were diagnosed on the basis of history taking, clinical examination and ultrasonography assessment. Smokers were instructed to avoid smoking 4 weeks preoperatively.

### Operative technique

**Fig. 1:**
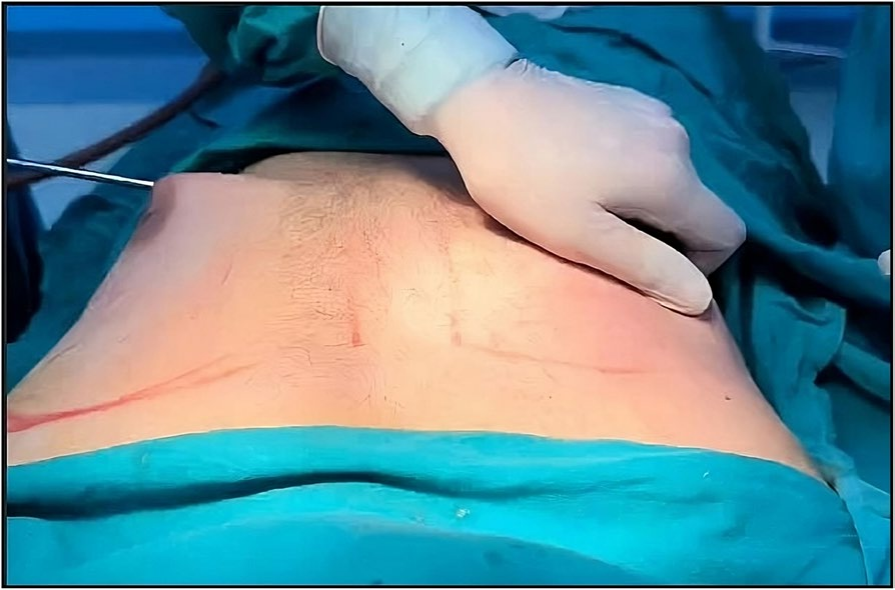
Cross-chest liposuction

Surgery was conducted under general anesthesia with the patient placed in the supine position and arms abducted 90^○^ at the chest. The following steps were performed on both affected breasts:

1. Single minimal skin incision: The whole surgical procedure was achieved through a minimal 1-cm stab incision made at the inferolateral areolar border. The incision was used for injection of tumescent solution, liposuction and gland excision.
2. Injection of tumescent solution: A tumescent solution containing 2% lidocaine (20 ml) and epinephrine (1 ml) in 1000 ml of normal saline was injected through the superficial and deep layers of fibroglandular tissue. To allow maximal infiltration of the tumescent solution, liposuction was started after 30 min of injection.
3. Cross-chest liposuction: Cross-chest liposuction is achieved through the same incision using 4-mm cannula for the deep layers and 3-mm cannula for the superficial layers. Cannulas were used for liposuction of the lateral chest on the ipsilateral breast and the whole contralateral breast except for its lateral side (Fig. 1).
4. Gland excision: After liposuction, attachments of glandular tissue were dissected and divided and the residual fibro-glandular tissue was extracted through the same incision. When needed, the incision was slightly extended to allow smooth extraction of the gland. To preserve the contour of nipple areola complex and avoid its depression, enough amount of sub-areolar tissue was left in place. Finally, incision was closed in layers.

### Postoperative care

Appropriate dressing was applied. Oral antibiotics were given for 1 week and patients were instructed to use a surgical corset for one month to reduce the risk of hematoma and seroma formation.

### Outcome assessment and follow up

Operative outcome assessment included aesthetic assessment and observation for postoperative complications. Patients were followed weekly in the first month and monthly later for an average follow up duration of 7.3 ± 1.6 months (range: 6–9 months). Aesthetic assessment was performed 6 months postoperatively for all patients while they were observed for any possible complications (e.g. hematoma and hypopigmented scar) until the last follow up visit.

Aesthetic assessment was performed by both patients and surgeons using a five-point Likert scale from 1 to 5 (1: very dissatisfied, 2: dissatisfied, 3: fair, 4: satisfied, and 5: very satisfied). Evaluated aesthetic aspects included breast symmetry, nipple and areola shape, projection of the nipple-areolar complex (NAC), contour regularity, and overall appearance [15]. Every patient was assessed by two independent surgeons who were blinded to the study technique. When assessments of the two observers varied, the lower score was used to make sure that results are not overestimated. Aesthetic outcome assessment was supervised by an independent researcher who wasn’t aware of the surgical technique.

### Statistical analysis

Data obtained from the present study were expressed as mean and standard deviation (SD) or number and percent. Comparison between numerical data was achieved using student t test while correlation analysis was achieved using Pearson’s correlation coefficient. All statistical calculations were computed using Statistical Package of Social Sciences version 27 (IBM, USA) with *p* value less than 0.05 considered statistically significant.

## Results

The present study was conducted on 30 patients with grade I/II gynecomastia and age of 29.9 ± 7.3 years. They had a body mass index (BMI) of 25.6 ± 2.7 kg/m^2^ and 3 (10.0%) of them were obese (body mass index > 30 kg/m^2^). Nine patients (30.0%) were smokers. The reported liposuction amount was 337.0 ± 43.8 ml while the amount of breast tissue removed was 53.5 ± 12.8 g. Liposuction duration was 20.2 ± 2.1 min while breast surgery duration was 15.4 ± 1.1 min and the total surgery duration was 35.7 ± 2.4 min (Table 1).

**Table 1:** Baseline and operative data (*n* = 30)

**Age** (years) mean ± SD	29.9 ± 7.3
**Body mass index** (Kg/m^2^) mean ± SD	25.6 ± 2.7
**Obesity** n (%)	3 (10.0)
**Smoking** n (%)	9 (30.0)
**Liposuction amount** (ml) mean ± SD	337.0 ± 43.8
**Breast tissue removed** (g) mean ± SD	53.5 ± 12.8
**Liposuction duration** (min.) mean ± SD	20.2 ± 2.1
**Breast surgery duration** (min.) mean ± SD	15.4 ± 1.1
**Total duration** (min.) mean ± SD	35.7 ± 2.4

**Fig. 2:**
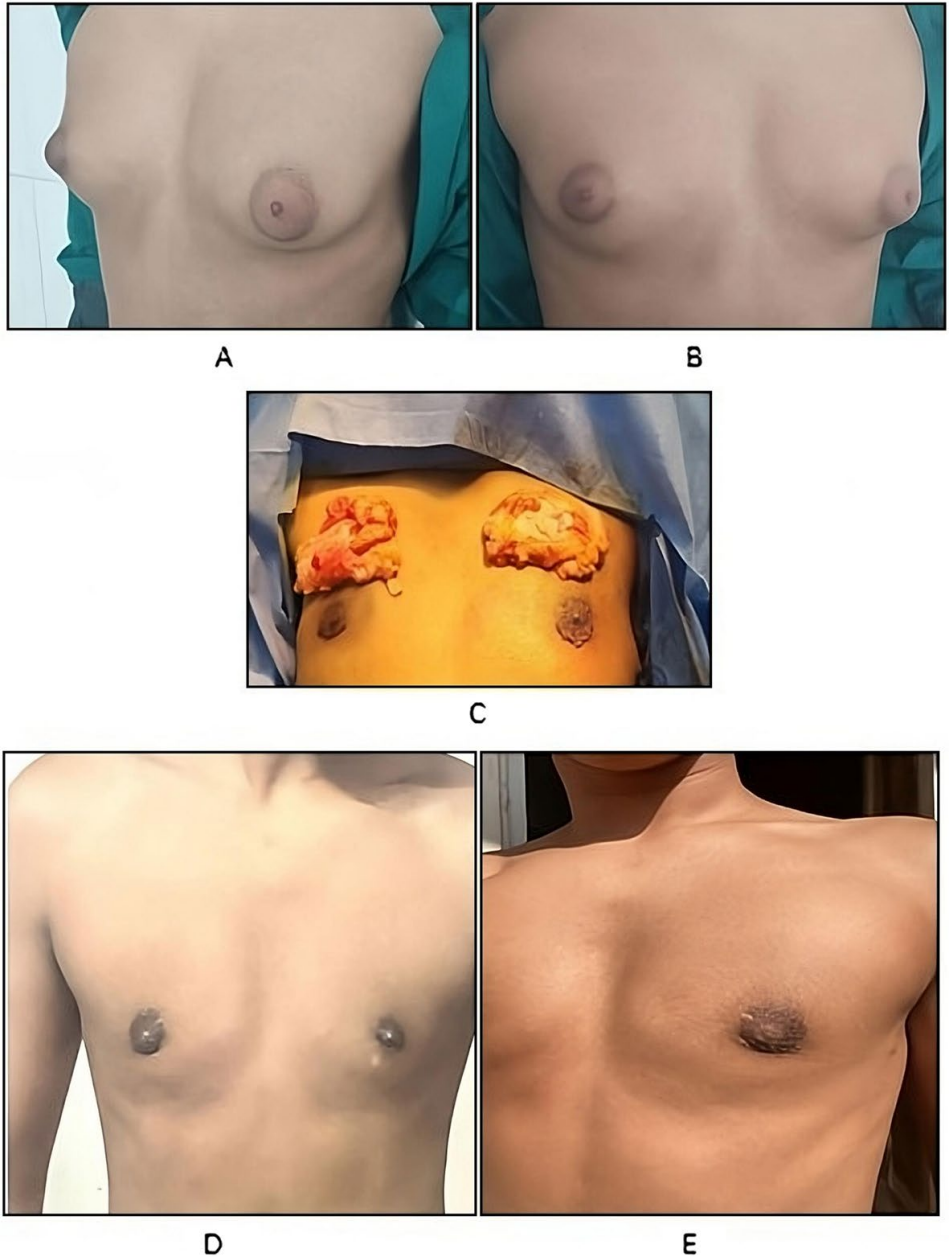
Preoperative (**A**, **B**) images, excised gland (**C**) and postoperative images (**D**, **E**)

**Fig. 3:**
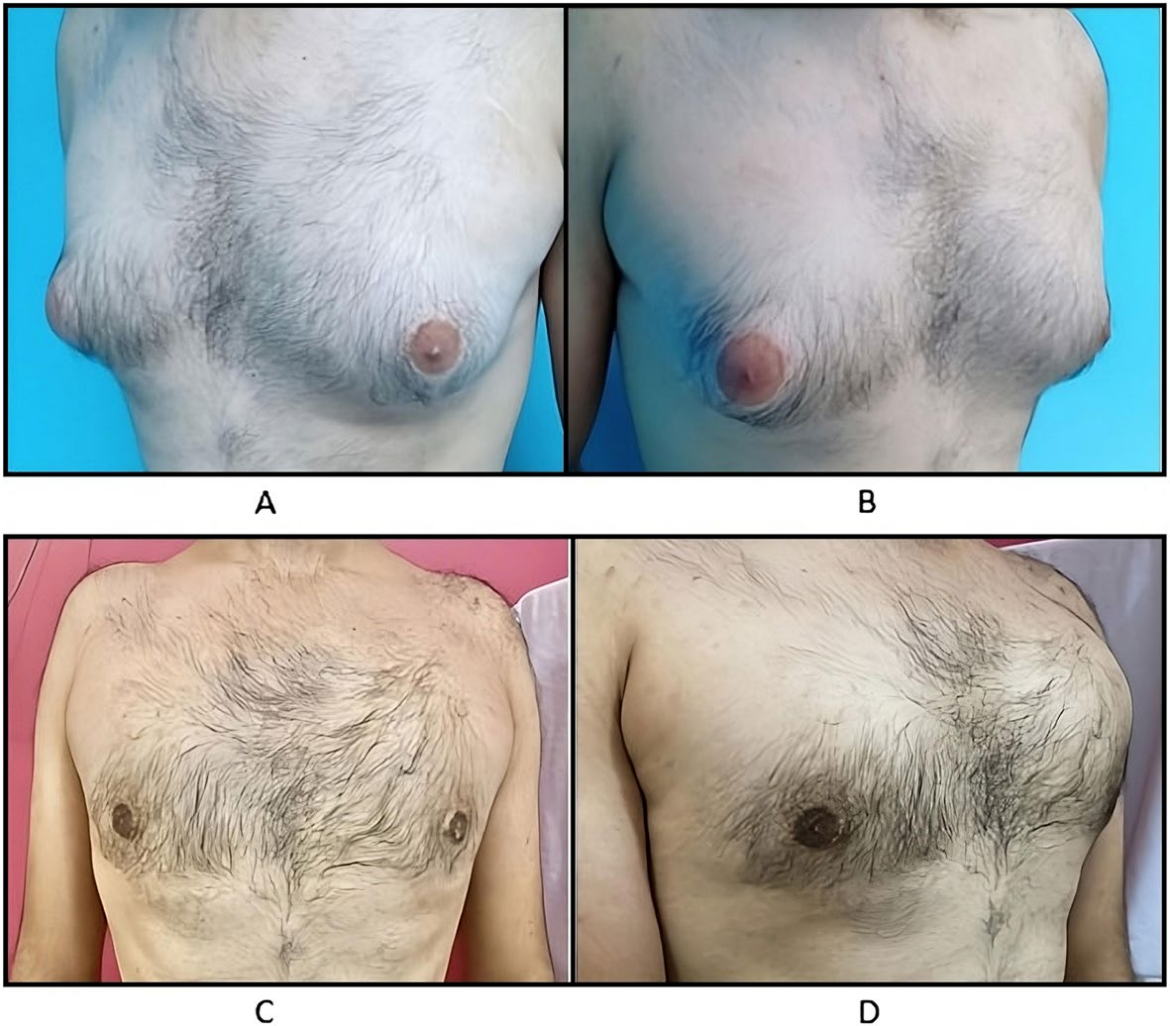
Preoperative (**A**, **B**) and postoperative (**C**, **D**) images

Postoperative complications included hematoma (3.3%) and very minimal hypopigmented scar (6.7%). Hematoma was effectively managed using standard measures. Both complications didn’t affect the aesthetic outcome. Patients were followed for an average duration of 7.3 ± 1.6 months. Patient-reported scores for breast symmetry, nipple and areola shape, projection of the NAC, contour regularity and overall appearance were 4.2 ± 0.4, 4.3 ± 0.5, 4.3 ± 0.4, 4.1 ± 0.3 and 4.4 ± 0.5 respectively while surgeon-reported scores for breast symmetry, nipple and areola shape, projection of the nipple-areolar complex, contour regularity and overall appearance were 4.5 ± 0.5, 4.4 ± 0.5, 4.4 ± 0.5, 4.2 ± 0.4 and 4.1 ± 0.3 respectively (Table 2; Figs. 2 and 3). None of the studied patients required surgical revision and nipple sensitivity was preserved in all patients.

**Table 2:** Outcome parameters in the studied patients (*n *= 30)

**Follow up duration** (months) mean ± SD	7.3 ± 1.6
**Operative complications** n (%)	
Hematoma	1 (3.3)
Hypopigmented scar	2 (6.7)
**Patient-reported outcome** mean ± SD	
Breast symmetry	4.2 ± 0.4
Nipple and areola shape	4.3 ± 0.5
Projection of the nipple-areolar complex	4.3 ± 0.4
Contour regularity	4.1 ± 0.3
Overall appearance	4.4 ± 0.5
**Surgeon-reported outcome** mean ± SD	
Breast symmetry	4.5 ± 0.5
Nipple and areola shape	4.4 ± 0.5
Projection of the nipple-areolar complex	4.4 ± 0.5
Contour regularity	4.2 ± 0.4
Overall appearance	4.1 ± 0.3

Comparison between patients classified according to their age (< mean and ≥ mean) or BMI (< 25 kg/m^2^ and ≥ 25 kg/m^2^) revealed no statistically significant differences regarding patient and surgeon reported outcomes (Table 3). Correlation analysis identified significant correlation between patient and surgeon reported outcomes in different aesthetic assessment parameters including breast symmetry (*r* = 0.39, *p* = 0.031), nipple and areola shape (r = 0.6, *p* < 0.001), projection of NAC (*r* = 0.59, p < 0.001), contour regularity (*r* = 0.48, *p* = 0.007) and overall appearance (*r* = 0.44, p = 0.015) (Table 4).

**Table 3:** Relation between patient and surgeon reported outcome and patients’ age and body mass index

	**Age** (years)		p value	**BMI** (Kg/m^2^)		*p* value
	< mean *N* = 17	≥ mean *N* = 13		< 25 *N* = 19	≥ 25 *N* = 11	
**Patient-reported outcome** mean ± SD						
Breast symmetry	4.3 ± 0.5	4.2 ± 0.4	0.38	4.3 ± 0.5	4.2 ± 0.4	0.63
Nipple and areola shape	4.2 ± 0.4	4.4 ± 0.5	0.39	4.2 ± 0.4	4.5 ± 0.5	0.2
Projection of NAC	4.3 ± 0.5	4.2 ± 0.4	0.71	4.3 ± 0.5	4.2 ± 0.4	0.44
Contour regularity	4.1 ± 0.2	4.2 ± 0.4	0.22	4.2 ± 0.4	4.0 ± 0.0	0.4
Overall appearance	4.3 ± 0.5	4.5 ± 0.5	0.36	4.4 ± 0.5	4.4 ± 0.5	0.98
**Surgeon-reported outcome** mean ± SD						
Breast symmetry	4.5 ± 0.5	4.5 ± 0.5	0.72	4.6 ± 0.5	4.3 ± 0.5	0.32
Nipple and areola shape	4.4 ± 0.5	4.5 ± 0.5	0.79	4.4 ± 0.5	4.5 ± 0.5	0.86
Projection of the NAC	4.4 ± 0.5	4.5 ± 0.5	0.56	4.4 ± 0.5	4.5 ± 0.5	0.66
Contour regularity	4.3 ± 0.5	4.2 ± 0.4	0.39	4.3 ± 0.5	4.1 ± 0.3	0.13
Overall appearance	4.1 ± 0.2	4.2 ± 0.4	0.41	4.1 ± 0.3	4.1 ± 0.3	0.9

*NAC* Nipple areola complex

**Table 4:** Correlation between patient and surgeon reported outcomes

Surgeon reported outcome	Patient reported outcome									
	Breast symmetry		Nipple and areola shape		Projection of NAC		Contour regularity		Overall appearance	
	r	p	r	p	r	p	r	p	r	p
Breast symmetry	0.39	0.031	-	-	-	-	-	-	-	-
Nipple and areola shape	-	-	0.6	< 0.001	-	-	-	-	-	-
Projection of NAC	-	-	-	-	0.59	< 0.001	-	-	-	-
Contour regularity	-	-	-	-	-	-	0.48	0.007	-	-
Overall appearance	-	-	-	-	-	-	-	-	0.44	0.015

*NAC* Nipple areola complex

## Discussion

The present study aimed to assess the clinical outcome of a minimally invasive approach for management of GI/II gynecomastia. This approach combines cross-chest liposuction and gland excision through a single minimal incision at the inferolateral areolar border. Patients and independent surgeons were highly satisfied with the aesthetic outcome.

The present technique provides two main advantages for patients and surgeons. First, the technique is completely performed through one minimal incision which is related to better aesthetic outcome and shorter surgical duration without need of wound drainage. Second, use of cross-chest liposuction enhances removal of the peri-glandular fatty tissue which facilities proper identification and removal of the gland.

In fact, the recent years had witnessed increasing trend towards use of minimally invasive approaches for management of early-stage gynecomastia. The study of Xia et al. [11] used enhanced liposuction followed by open resection of the gland using the pull-through and bottom-up technique. In another report, Adhikari [16] used liposuction and minimal 5–7 mm nipple incision for gland extraction. Likewise, Shang et al. [17] used a combination of endoscopic subcutaneous mastectomy and liposuction for treatment of gynecomastia. In comparison to our study, the authors used two separate incisions for liposuction and gland removal.

In other studies, however, single incision was used. For example, Asal et al. [18] used liposuction and port site nipple sparing mastectomy with good aesthetic outcome while the study of Jian et al. [19] used single-port endoscopic mastectomy via the lateral chest approach for treatment of grade II gynecomastia. In comparison, the present study incision is located at the areolar margin which may provide better aesthetic appearance than provided by other incisions.

The reported average operative duration in the present study is about 35 min which is noticeably shorter than the 45 min reported by Asal et al. [18], the 86 min reported by Shang et al. [17], the 87 min reported by Jian et al. [19] and the 110 min reported by Adhikari [16]. The shorter operative duration is potentially linked to lower operative burden and costs.

Operated patients in this study experienced only minimal complications including postoperative hematoma and hypopigmented scar which didn’t affect the aesthetic outcome and none of them required surgical revision. In contrast, reported complications in the study of Xia et al. [11] included hematoma, scar hypertrophy, residual gland tissue and surgical revision while in the study of Adhikari [16], the main complications included hematoma and nipple hypopigmentation. Cross-chest liposuction in the present study served to improve the access of liposuction cannulas and enhance the quality of the process without the need of additional skin incisions [20–22].

Interestingly, both patient and surgeon clinical outcomes were comparable regardless patients age and BMI. Also, it was noted that patient and surgeon reported outcome were found to be well correlated in all aesthetic assessment aspects. These findings reflect the consistent outcome assessment across different patients’ subgroups.

### Limitations

Findings of the present study may be limited by the small sample size, lack of control group and relatively short follow up period.

## Conclusions

In conclusion, the present study found that the combination of cross-chest liposuction and gland excision through a single minimal incision at the inferolateral areolar border provides good and satisfactory aesthetic outcomes for patients with grade I/II gynecomastia.

## Data Availability

Data will be available from the corresponding author upon reasonable reques
